# Awn Stem-Derived High-Activity Free-Metal Porous Carbon for Oxidation Reduction

**DOI:** 10.3390/molecules26196071

**Published:** 2021-10-08

**Authors:** Qingyun Zhao, Shikai Wen, Junhua Hou

**Affiliations:** 1 School of Physics and Information Engineering, Shanxi Normal University, No.1 Gongyuan Road, Yaodu District, Linfen 041004, China; sxlfzqy@sohu.com (Q.Z.); 218110019@stu.sxnu.edu.cn (S.W.); 2 Modern College of Humanities and Sciences, Shanxi Normal University, No. 657 Jiefang East Road, Yaodu District, Linfen 041000, China; 3 Extreme Optical Collaborative Innovation Center, Shanxi University, No. 92, Wucheng Road, Xiaodian District, Taiyuan 030006, China

**Keywords:** biomass carbon, oxygen reduction reaction, N, S co-doped, catalyst

## Abstract

Designing oxygen reduction reaction (ORR) catalysts with excellent performance has far-reaching significance. In this work, a high-activity biomass free-metal carbon catalyst with N and S co-doped was successfully prepared by using the KOH activated awn stem powder as the precursor with organic matter pore-forming doping technology, which is named TAAS. The content of pyridine nitrogen groups accounts for up to 36% of the total nitrogen content, and a rich pore structure is formed on the surface and inside, which are considered as the potential active centers of ORR. The results show that the specific surface area of TAAS reaches 191.04 m^2^/g, which effectively increases the active sites of the catalyst, and the initial potential and half slope potential are as high as 0.90 and 0.76 V vs. RHE, respectively. This study provides a low-cost, environmentally friendly and feasible strategy for the conversion of low-value agricultural and forestry wastes into high value-added products to promote sustainable development of energy and the environment.

## 1. Introduction

Oxygen reduction reaction (ORR) is a particularly important part of the new energy storage and conversion devices [1,2,3]. However, excessive overpotential and slow kinetics of ORR at the cathode are the main challenges that the realization of a wide range of commercial applications of fuel cells face [4,5,6,7]. At present, the Pt-based catalyst, which is considered the most efficient catalyst, has been limited in commercialization due to its finite global reserves, high price and poor tolerance. Thus, an inevitable trend to find substitutes for Pt-based catalyst results [8]. In recent years, biomass carbon materials have been favored by scientists for their advantages such as wide sources, low price, controllable pore structure, high conductivity, high stability and tolerance [9,10,11]. As a result, converting biomass into carbon-based catalysts has become an effective strategy for achieving high value added to the catalyst and reducing catalyst costs [12].

At present, huge amounts of non-precious metal catalysts, transition metal oxides, nitrides and non-metal catalysts have been developed. Specifically, non-metallic heteroatoms (N, S, P, B, etc.) doping can effectively change SP^2^ carbon’s self-selected distribution and charge, resulting in uneven charge distribution and the increase in defects, which is conducive to the adsorption of O_2_ and effective improvement of the activity of ORR catalyst [13,14,15,16]. The increase in defects can not only directly serve as active sites but also help to change the electronic structure of the catalyst surface and improve the catalytic activity [17]. As research shows, the doping of N atoms into carbon materials can effectively enhance mass transfer and enrich active sites. Moreover, multi-element co-doping also benefits the improvement of catalyst activity. Therefore, the controllability of pore size and the high doping rate of N element are indispensable for synergistically improving catalyst activity [18,19,20,21]. However, since the content of N element in biomass carbon is limited, it is essential to choose suitable biomass precursors and appropriate preparation processes to prepare porous biomass carbon materials with high catalytic activity enriched in active sites.

Herein, we used awn stem which is a handy plant straw in Shanxi province as the precursor of carbon material. At the same time, N and S co-doping in the catalyst was successfully realized through KOH activation and organic pore-making doping technology, which effectively improved the performance of the catalyst [22,23]. The surface morphology and structural characteristics of the catalyst were characterized by SEM, TEM, BET, Raman, FT-IR and XPS, and the mechanism of catalyst activity improvement was further explained. It was found that after activation and organic pore formation, the initial potential and half-wave potential of TAAS reached 0.97 and 0.76 eV vs. RHE, respectively, which is caused by the synergy of the doping of N and S and the increase in specific surface area and defects. This work provides a simple, low-cost and easily popularized method for the conversion of agricultural and forestry wastes into high value-added products.

## 2. Experimental Section

### 2.1. Materials

The awn stems are from the Lvliang region of Shanxi province, China. Trithiocyanuric acid (analytical purity) was purchased by Sinopharm Holding Chemical Co, Ltd. (Shanghai, China), and no purification treatment was performed before use.

### 2.2. Materials Synthesis

The awn stems collected in Lvliang were washed with deionized water and dried at 60 °C. A pulverizer was used to crush the stems into powder for later use. The amount of 5 g of awn stems powder with a particle size of less than 200 mesh was placed into 1 M KOH solution at 60 °C for 48 h in order to activate them. The powder was then wash with deionized water until it was neutral and dried at 60 °C. One gram of activated awn stem powder and trithiocyanuric acid was mixed in 50 mL of deionized water at a mass ratio of 1:3, stirred at room temperature for 2 h and dried at 60 °C. The dried sample was incubated at 550 °C for 4 h in an N_2_ atmosphere and heated to 900 °C for 2 h at the heating rate of 5 °C/min in order to obtain the catalyst marked as TAAS. In order to study the influence of KOH activation and trithiocyanuric acid on the catalysts, the same carbonization method was used to prepare the catalysts with the untreated and activated awn stem powder, respectively, labeled AS and AAS (Table S1).

### 2.3. Structural Characterization

The morphology and structure of all catalysts were observed using filed emission scanning electron microscope at under 10 KV (SEM, VP-EVO, MA-10, Carl-Zeiss, UK) and transmission electron microscope (TEM, JEOL JEm-2100F). X-ray powder diffraction was used to evaluate the crystal structure of the catalysts with Cu-Kα radiation in the 2θ range of 10° to 80°. The physical and chemical properties of the samples were characterized by Fourier transform infrared spectroscopy (FT-IR, Bio-Rad FTIR spectrometer FTS165) in the wavenumber range of 100~4000 cm^−1^. The X-ray photoelectron spectroscopy (XPS) of the catalysts surface analysis were measured by AMICUS electron spectrometer on SHIMADZU with 300 W Al Kα radiation. The specific surface area and pore size distribution are obtained by using the Micromeritics Ga 30093-2901 U.S.A. instrument. An electrochemical workstation (Autolab) was used for electrochemical testing of all catalysts.

## 3. Results and Discussion

**Figure 1:**
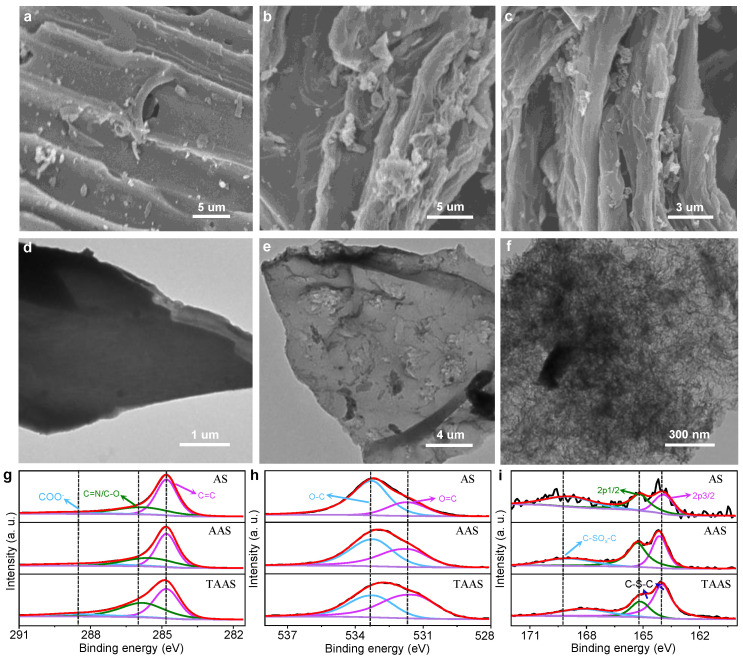
SEM images of AS (**a**), AAS (**b**) and TAAS (**c**). TEM images of AS (**d**), AAS (**e**) and TAAS (**f**). High-resolution XPS spectrum for the C 1s (**g**), O 1s (**h**) and S 2p (**i**) peaks of AS, AAS and TAAS.

As shown in Figure 1, in order to observe the changes of surface morphology and microstructure, all the catalysts were characterized by field emission scanning electron microscopy and transmission electron microscopy. As shown in Figure 1a–c, compared with the pore structure of AS (a) and AAS (b), the SEM results of the catalyst TAAS (c) after KOH activation and organic pore formation and doping have obviously uneven lamella-like structure. Figure 1d–f are the TEM scanning results of AS, AAS and TAAS, respectively, which are consistent with the SEM results. The directly carbonized catalyst AS presents a smooth lamellar structure, while the activated catalyst AAS has a clear pore structure on the surface. The result of TAAS presents a flocculent structure, indicating that a rich pore structure has formed on its surface and on the inside, which provides a basis for the increase in active sites of the catalyst and the enhancement of catalytic activity [24]. Therefore, the application of KOH activation and organic pore-forming doping technology can effectively promote the increase in surface and internal pore structure of the rod, which improves the accessibility of active sites and the diffusion rate of ions during the electrochemical reaction of the catalyst. All of these caused the increase in activity of the catalyst [25,26,27].

In Figure 1g, the high-resolution spectrum of C 1s can be deconvoluted into three component fronts centered at 284.7, 285.7 and 288.1 eV, corresponding to C=C (sp^2^) in graphene, C-N/C-O and C=O, respectively. The results showed that the N element was successfully doped into the carbon network. Similarly, the O 1s high-resolution spectra of all catalysts (Figure 1h) showed two characteristic peaks at the positions of 531.6 and 533.3 eV. It can be observed that the peak at 531.6 eV is related to the carboxyl group O=C in the carboxylate, while the peak at 533.3eV corresponds to O-C. In addition, as shown in Figure 1i, the high-resolution S 2p spectra of all catalysts show two broad regions. The region of 163–166 eV represents aromatic sulfides. Due to spin coupling, S 2p^3/2^ and S 2p^1/2^ centered at 164.1 and 165.1 eV correspond to C-S-C and C=S, respectively. The peak (186.5 eV) in the higher energy region is consistent with the C-SOx-C group [28,29].

**Figure 2:**
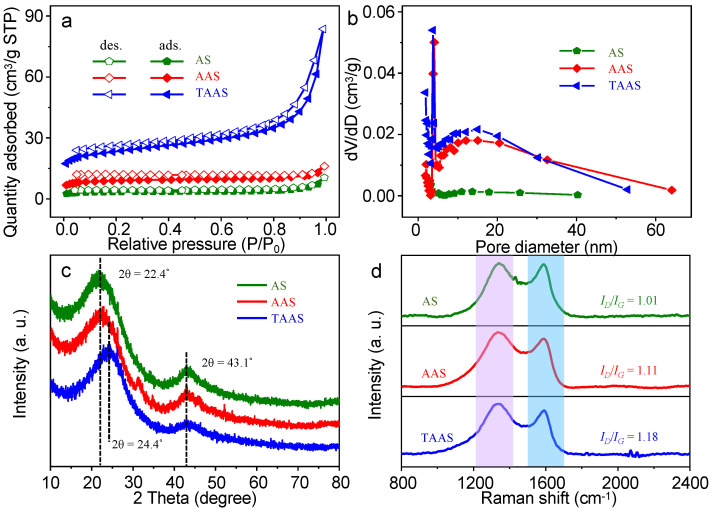
N_2_ adsorption-desorption isotherms (**a**) and corresponding pore size distribution diagrams (**b**) of AS, AAS and TAAS. XRD patterns (**c**) and Raman spectra (**d**) of all catalysts.

As we all know, specific surface area and pore structure are two important factors that affect the catalytic performance of catalysts [30]. Therefore, we used Brunauer–Emmett–Teller (BET) to characterize all catalysts. As reflected in Figure 2a, from the N_2_-adsorption and desorption isotherms of all catalysts, it can be observed that the curves of AS, AAS and TAAS all show typical type IV isotherms, which demonstrates their mesoporous structure. The results show that the specific surface area of TAAS (191.04 m^2^/g) increases significantly compared to AAS (48.86 m^2^/g) and AS (21.38 m^2^/g), which is consistent with the test results of SEM and TEM. It is worth noting that, as shown in Table S2, the pore volume of TAAS is 0.13 cm^3^/g, while the pore volumes of AS and AAS are only 0.016 and 0.024 cm^3^/g, respectively. This is the result of the etching effect of KOH on the awn stem powder during the activation process and trithiocyanuric acid after high temperature gasification and pyrolysis consuming part of the unorganized carbon during the preparation of carbon materials, thereby promoting the formation of the pore structure and increasing the specific surface area of rich carbon materials. In addition, as shown in Figure 2b, the BJH pore size distribution results show that the pore sizes of all catalysts are mainly distributed between 5 and 30 nm, indicating that AS, AAS and TAAS all include mesopores, which is consistent with the results of the N_2_-adsorption and desorption isotherm. The high specific surface area and abundant pore structure play important roles in the increase in catalyst active sites, the improvement of the ion transport rate during the ORR process and the improvement of catalytic activity [30].

XRD patterns are used to characterize the crystal structures of AS, AAS and TAAS. It can be observed from Figure 2c that AS and AAS both have peaks centering at 2θ ≈ 22.4°, and TAAS has a peak center at 2θ ≈ 24.4°, all of which corresponds to the typical graphite (002) planes. All samples have peaks centering at 2θ ≈ 43.1°, which corresponds to the graphite phases in the N-dope biochar (101) [31,32]. It is worth noting that the (002) crystal plane of the catalyst TAAS has an obvious tendency to move to the right, and the diffraction peak becomes wider, which is attributed to the increase in interplanar spacing and lattice defects caused by heteroatom doping. In addition, the result also indicates that the degree of graphitization of the catalyst TAAS is reduced [33]. Therefore, since the doping of heteroatoms helps to suppress continuous sp^2^-C plane conformation of the carbon material, it can render the carbon structure of the catalyst more disordered.

The Raman diagram further analyzes the degree of graphitization and defects of the catalyst. As is reflected by the Figure 2d, all the catalysts have two broad characteristic peaks at the center of about 1335 cm^−1^ and 1580 cm^−1^, corresponding to the D and G peaks, respectively [34]. The D peak represents the lattice defects and disorder of the carbon materials, and the G peak is related to in-plane stretching vibrations of the sp^2^ hybridization of carbon materials. The ratio of peak D to peak G (*I_D_*/*I_G_*) is an important indicator for judging the degree of graphitization and defect of carbon materials [35,36]. The larger the ratio, the lower the degree of graphitization and the higher the defect degree of the corresponding material. The *I_D_*/*I_G_* ratios of AS, AAS and TAAS are 1.01, 1.11 and 1.18, respectively. Compared with AS and AAS, the ratio of TAAS has increased dramatically, indicating that after KOH activation and trithiocyanuric acid pore formation and doping, the degree of graphitization of TAAS is distinctly reduced, and the degree of defects increases, which are consistent with the XRD test results. It is proved that the doping of nitrogen significantly increases the defect structures and active sites of the catalyst, which results in an increase in the activity of the catalysts.

**Figure 3:**
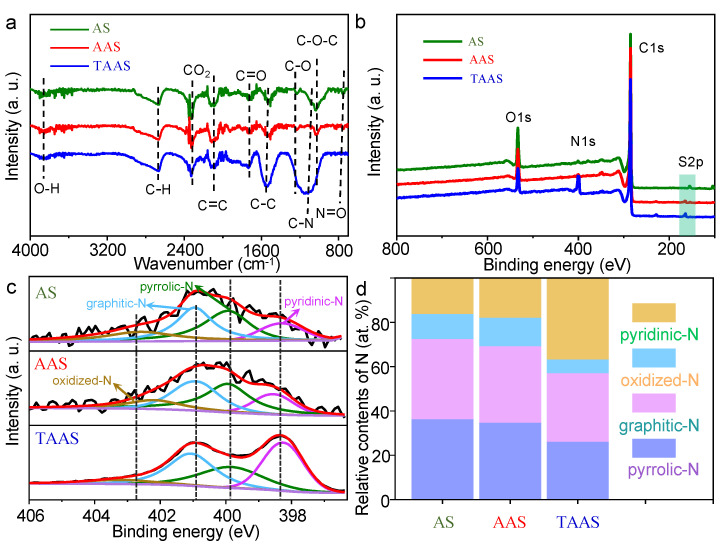
FT-IR diagram of AS, AAS and TAAS (**a**). XPS spectrum (**b**), high-resolution XPS spectrum of N 1s (**c**) and 3D bar graphs of the relative content of nitrogen species on the surface of AS, AAS and TAAS (**d**).

Typically, the crown energy group and chemical composition of the catalyst are determined by Fourier infrared spectroscopy and X-ray electron spectroscopy [37,38]. As shown in Figure 3a, AS, AAS and TAAS basically show the same absorption peak at the same position, indicating that the functional groups of all catalysts are basically the same. All catalysts showed obvious absorption peaks near 3865 and 2665 cm^−1^, corresponding to O-H and C-H stretching vibrations, respectively. The absorption peaks near 2324 and 2110 cm^−1^ can be attributed to the stretching vibration of CO_2_ and C=C. The absorption peaks near 1723 and 1525 cm^−1^ are related to C=O and C-C stretching vibrations. In addition, the absorption peaks near 1223 and 1026 cm^−1^ are caused by stretching vibrations of C-O and C-O-C, and the absorption peaks of TAAS at these two positions almost disappear, indicating that KOH activation destroys the fiber structure in TAAS, thus forming abundant pore structure, which is consistent with the obvious increase in the specific surface area of TAAS relative to AS. Compared with AS and AAS, TAAS has obvious absorption peaks near 1126 and 828 cm^−1^, which are the results of C-N and N=O stretching vibration, respectively. The results demonstrate that N atoms were successfully introduced into the catalyst TAAS.

X-ray electron spectroscopy (XPS) data were used to further analyze element composition and bonding configuration of the catalyst [39]. As shown in Figure 3b, in terms of the XPS measurement spectrum, the catalysts presented S 2p, C 1s, N 1s and O 1s peaks centered at 164, 285, 400 and 532 eV, respectively. According to Table S3, the element content of AS and AAS are almost the same, indicating that the KOH activation process did not change the element composition of the catalyst. Most notably, compared to the nitrogen content of AS (1.47 at%) and AAS (1.34 at%), TAAS significantly increased to 9.84 at%. In addition, the sulfur content of TAAS increased from 0.22 at% (AS) to 1.4 at%, indicating that trithiocyanuric acid acts as a donor of N and S during the pyrolysis process, providing active sites and pseudo capacitance for highly active catalysts. In Figure 3c, the high-resolution N 1s spectrum of the catalyst has peaks centered at 398.3, 499.8, 401.0 and 402.5 eV corresponding to pyridine-N, pyrrole-N, graphite-N and oxide-N, respectively. Pyridine-N and graphite-N are considered to be the most useful structures for improving the ORR activity of the catalyst in nitrogen doping. Figure 3d shows the different nitrogen content of all catalysts. The content of graphite-N is almost the same, while the content of pyridine-N in TAAS increased dramatically from 16% to 36%, which is beneficial to the improvement of TAAS catalytic activity.

**Figure 4:**
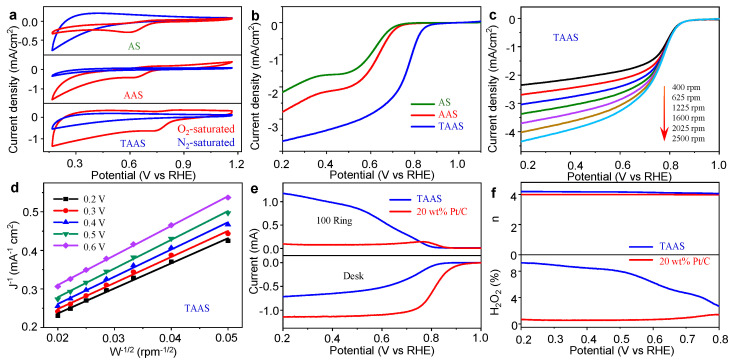
CV curves (**a**) of all catalysts in O_2_ or N_2_-saturated 0.1 M KOH solution at room temperature and the scanning rate of 50 mV/s. The LSV curves of AS, AAS and TAAS at the electrode rotation rate of 1600 rpm and the scanning rate of 10 mV/s (**b**). The LSV curve (**c**) of TAAS at different speeds from 400 to 2500 rpm coincide with the K-L diagram (**d**) of TAAS from 0.2 to 0.6 V. The RRDE linear sweep voltammogram of TAAS and 20 wt% Pt/C in O_2_-saturated 0.1 M KOH at the electrode rotation rate and the scanning rate are 1600 rpm and 5 mV/s, respectively (**e**). Electron transfer number n (**up**) and H_2_O_2_ yield (**down**) calculated from the RRDE measurement results of TAAS and 20 wt% Pt/C (**f**).

The ORR catalytic performance of all catalysts was tested with Autolab electrochemical workstation in a three-electrode system at room temperature. The ORR catalytic behavior of all catalysts was studied by cyclic voltammetry (CV), and these measurements were carried out at the scanning rate of 50 mV/s in the potential range of 0.2–1.2 V (vs. RHE). Figure 4a shows the cyclic voltammetry curves in 0.1 M O_2_-saturated KOH solution, and there are obvious cathodic peaks in the potential range of 0.6~0.8 V (vs. RHE), and the potential increases are in the order of AS < AAS < TAAS. In the N_2_-saturated KOH solution, TAAS showed a typical CV curve without a reduction peak. It shows that the ORR catalytic reaction only occurs at the electrode in the O_2_-saturated electrolyte. In order to further study the ORR catalytic performance of the catalyst, the LSV curves of all catalysts were recorded in the range of 0.2~1.2 V (vs. RHE) potential with the electrode speed of 1600 rpm, the scanning rate of 10 mV/s and the O_2_-saturated 0.1 M KOH electrolyte, as shown in Figure 4b. It is apparent from the figure that the initial potential of TAAS is 0.90 V vs. RHE, which is significantly higher than the potential of AS (0.71 V vs. RHE) and AAS (0.73 V vs. RHE), and the half-wave potential increases in the order of AS (0.62 V vs. RHE) < AAS (0.64 V vs. RHE) < TAAS (0.76 V vs. RHE). This can be attributed to the increase in TAAS specific surface area, surface and internal defects and pyridine-N content [40].

Figure 4c shows that the linear sweep voltammetry (LSV) curves of TAAS were tested by the rotating disk electrode (RDE) at different speeds from 400 to 2500 rpm and a scanning rate of 10 mV/s. Similarly, Figures S1a and S2a show the LSV curves of AS and AAS at different speeds, respectively. The results shows that the initial potential of the catalyst remains constant, while the current density increases with the enhancement of the rotation rate of the rotating disk electrode. This is due to the shrinkage of the diffusion distance between the O_2_ interface and the electrode. Based on the RDE test results, the ORR kinetic parameters and Koutecky–Levich (K-L) diagram are obtained through the K-L equations with the relationship between the reciprocal of the current density (J^−1^) and the reciprocal of the square root of the RDE rotation rate (ω^−1/2^). Figure 4d and Figures S1b and S2b, respectively, show the K-L diagram of catalysts TAAS, AS and AAS. The results suggest that J^−1^ and ω^−1/2^ present a linear relationship in the range of 0.2~0.6 V vs. RHE. In the K-L diagram, the fitted lines at different potential are parallel to each other, indicating that all the catalysts follow first-order reaction kinetics during the ORR reaction [41]. In addition, the number of electrons transferred per oxygen molecule of TAAS varies from 3.64 to 4.21 at different potentials, and the average number of electrons transferred is 3.95, which is slightly lower than the ideal value of four, showing that the four-electron transferred process is dominant in the ORR reaction process. Similarly, the average number of electrons transferred with respect to AAS and AS are 3.37 and 1.60, respectively. The results demonstrate that AAS follows the four-electron transferred pathway, and AS is dominated by the two-electron transferred process [42].

In order to further verify the electron transferred pathway of TAAS in the ORR reaction process, as shown in Figure 4f, we calculated the number of transferred electrons and H_2_O_2_ yield of TAAS and 20 wt% Pt/C during the ORR reaction by the test results of the rotating ring disk electrode (RRDE) in O_2_-saturated 0.1 M KOH electrolyte (Figure 4e). As observed from Figure 4e, both TAAS and 20 wt% Pt/C show lower ring current densities, indicating that both of them have higher catalytic activity, and the H_2_O_2_ content generated at the ring electrode is quite low. Based on the ring current data of RRDE (Figure 4f), according to the formula, it can be calculated that the H_2_O_2_ yields of TAAS and 20 wt% Pt/C were 9.27 and 0.67%, respectively. Similarly, based on the disk current data of RRDE, it is clear that the n value of TAAS is in the range of 4.05~4.18, which is slightly higher than the n value range of 3.97~3.98 of 20 wt% Pt/C. The results are consistent with those of the K-L spectrum, which further proves that TAAS follows the four-electron transferred pathway during the ORR reaction [43,44].

**Figure 5:**
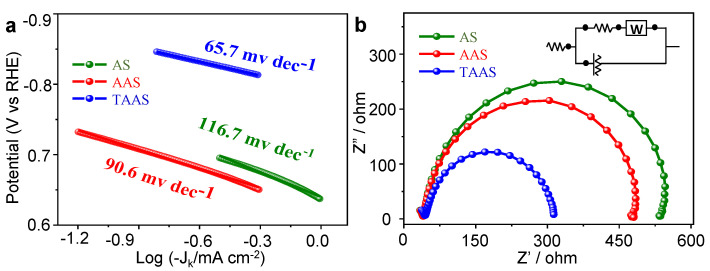
Tafel slope curve spectrum (**a**) and electrochemical impedance (EIS) spectrum (**b**) of AS, AAS and TAAS.

In order to further explore the ORR kinetics of the catalyst, we obtained the Tafel slope of all catalysts through the LSV curve at low potential. The smaller the Tafel slope value, the faster the electron transferred rate and the better the catalytic performance becomes. As shown in Figure 5a, the Tafel slopes of AS, AAS and TAAS decrease in the order of 116.7 mV dec^−1^ > 90.6 mV dec^−1^ > 65.7 mV dec^−1^, indicating that when there is closer contact between the electrolyte and the electrode, the faster the electron transferred rate becomes and higher catalytic activity during the ORR reaction of TAAS results [45]. In order to determine the charge transfer impedance at the cathode/electrolyte interface (R_ct_, high frequency semicircle, the lower the value and the faster the charge transfer), we performed electrochemical impedance spectroscopy (EIS) tests on all catalysts. Figure 5b shows the equivalent circuit diagram of impedance spectrum fitting (inset) and the Nyquist spectrum of all catalysts. It can be observed from Figure 5b that the R_ct_ value of TAAS is 390 Ω, while AS and AAS are 480 Ω and 550 Ω, respectively. Obviously, the value of TAAS is much lower, indicating that TAAS has faster charge transfer rate in the ORR reaction process. This can be attributed to the increase in specific surface areas of TAAS and its abundant pore structure, which effectively improves the contact area between the electrolyte and the electrode, which increases the charge transfer rate during the charge and discharge process. In addition, the co-doping of N and S atoms effectively improves the electrical conductivity of the material and the ORR catalytic performance of TAAS [46].

**Figure 6:**
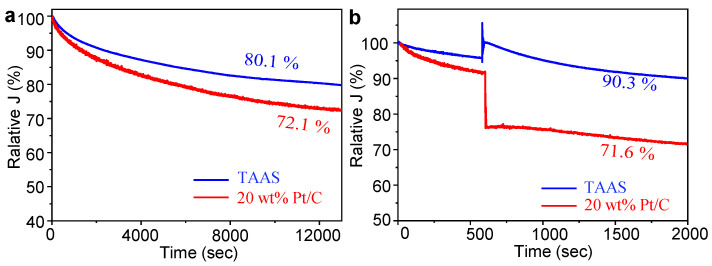
The stability curve of TAAS and 20 wt% Pt/C in O_2_-saturated 0.1 M KOH solution, RDE rotation rate of 1600 rpm, 15,000 s stability curve (**a**) and the methanol tolerance performance curve measured by current-time chronoamperometry (**b**).

As we all know, the oxidation of methanol on Pt-based catalysts is the main reason the stability of fuel cells is affected [47,48]. Therefore, long-term stability and methanol resistance are two important indicators for judging catalyst performance. The stability and methanol tolerance of TAAS and 20 wt% Pt are measured in the O_2_-saturated 0.1 M KOH solution at the RDE rotation rate of 1600 rpm and the potential of −0.3 V (vs. Ag/AgCl) by current-time chronoamperometry. As shown in Figure 6a, after 13,000 s, TAAS still maintains 80.1% of the initial current, which is significantly higher than 72.1% of 20 wt% Pt/C. To test the methanol tolerance of the catalyst, we quickly added 5 mL of methanol to the electrolyte at the 600th s, then the current density of 20 wt% Pt/C dropped sharply. At 2000 s, TAAS and 20 wt% Pt /C maintained the initial currents of 90.3% and 71.6%, respectively (Figure 6b). The results illustrate that long-term stability and methanol tolerance of TAAS are obviously better than those of 20 wt% Pt/C, which can meet the requirements of practical applications. These results are due to the synergistic effect of the increase in TAAS specific surface area and surface modification.

In summary, the reasons for the significant increase in the ORR catalytic activity of TAAS are as follows: (1) The increases in specific surface area and abundant pore structure provide more active sites for the catalyst, which is the material basis for the improvement of catalyst activity; (2) the surface and edge defects of the catalyst increase. They can be directly used as the active sites of the reaction. At the same time, it helps to improve the electronic structure of the catalyst surface, decrease reaction adsorption energy and effectively improve the catalytic performance of the catalyst. (3) N and S co-doping and the defects of the catalyst itself form new active sites. (4) The increase in the content of pyridine-N also remarkably improved catalyst activity. Therefore, the increase in TAAS catalytic activity is the result of the synergistic effect of the improvement of surface morphology, the optimization of the structure and the N and S co-doping of the catalyst.

## 4. Conclusions

In this study, the abundant, cheap, environmentally friendly and renewable awn stem is used as a precursor, and a simple, safe and easily operated organic pore-forming doping technology is employed to prepare a highly active ORR catalyst. The specific surface area of TAAS increased from 21.38 m^2^/g to 191.04 m^2^/g. This is the result of the etching effect of KOH on the awn stem and the co-doping of N and S, which effectively increased the defect and the number of active sites in the catalyst. In addition, the increase in pyridine-N content helped to form new active sites and improved the performance of the catalyst. The catalyst TAAS showed excellent catalytic activity during the ORR reaction. The initial potential and half-wave potential reached 0.86 and 0.76 V vs. RHE, respectively. It is worth noting that the stability and methanol resistance of TAAS were more superior to those of 20 wt% Pt/C. The work provides a low-cost, environmentally friendly and feasible strategy for the conversion of low-value agricultural and forestry waste into high value-added products to promote the sustainable exploitation and development of energy and the environment.

## Data Availability

Not applicable.

## Supplementary Materials

The following are available online, Figure S1: The LSV curves at different rotation rates from 400 to 2500 rpm of AS (a), corresponding to the K-L plots of AS from 0.2 to 0.6 V (b), respectively, Figure S2: The LSV curves at different rotation rates from 400 to 2500 rpm of AAS (a), corresponding to the K-L plots of AAS from 0.2 to 0.6 V (b), respectively, Table S1: Synthesis process of catalyst AS, AAS and TAAS., Table S2: Physical properties and the charge transfer impedance of AS, AAS and TAAS, Table S3: The content of C, N, O, and S elements and N configuration calculated of AS, AAS and TAAS from elemental analysis and XPS.

## Sample Availability

Samples of the compounds are available from the authors.
