# Supplementary material for: Awn Stem-Derived High-Activity Free-Metal Porous Carbon for Oxidation Reduction
Source: Molecules. 2021 Oct 8;26(19):6071. doi: 10.3390/molecules26196071 (PMC8512104; doi:10.3390/molecules26196071)
Supplement: Supplementary file 1 [file molecules-26-06071-s001.zip › molecules-1356333-supplementary.pdf]

Awn Stem-Derived High-Activity Free-Metal Porous Carbon for Oxidation Reduction

Qingyun Zhao [1,2], Shikai Wen [1,2] and Junhua Hou [1,2,3,*]

[1] School of Physics and Information Engineering, Shanxi Normal University, No.1 Gongyuan Road, Yaodu District, Linfen 041004, China; sxlfzqy@sohu.com (Q.Z.); 218110019@stu.sxnu.edu.cn (S.W.)
[2] Modern College of Humanities and Sciences, Shanxi Normal University, No.657 Jiefang East Road, Yaodu District, Linfen 041000, China
[3] Extreme optical Collaborative Innovation Center, Shanxi University, No. 92, Wucheng Road, Xiaodian District, Taiyuan 030006, China
[*] Correspondence: jhhou@126.com or jhhou@sxnu.edu.cn;
Tel.: +86-357-2051395

Electrochemical measurements

All ORR performance was measured with a conventional three-electrode system at room temperature, wherein the reference electrode is an Ag/AgCl electrode (saturated KCl solution), the contrast electrode is a graphite bar and the working electrode is rotating the disc electrode (Glass carbon electrode, GC, d = 5mm). Before the experiment, graphite electrode and glass disc rotating disk (RDE, diameter = 5 mm, area = 0.1963 cm^2) were placed in 0.05mm of aluminum powder, then cleaned in an ultrasonic instrument, and finally washed with deionized water for a few minutes.

The ink preparation method is 5mg of catalyst, 50 μL of Nafion solution, 250 μL of isopropanol and 700 μL of deionized water were mixed in a 1.5mL centrifugation tube. The tube is first placed in the ultrasonic instrument for 1 hour to forming a homogeneously mixed catalytic ink. Finally, then evenly disperse 10 μL of suspension droplets on the electrode surface and dry naturally (the average catalyst load was 0.25 mg cm^{-2}). The 20wt % Pt/C electrode was prepared by the same method.

ORR electrochemical tests include circulating voltammetry (CV), linear scanning voltage (LSV) and current-time (I-t) chronoamperes with rotating disk electrode (RDE)

and the Autolab electrochemical work station. Convert all potentials to the potential of standard reversible hydrogen electrodes (RHE) by the following equation (equation (1)):

$$E_{（RHE）} = E_{(Ag/AgCl)} + E^{\theta}_{(Ag/AgCl))} + 0.059pH \qquad (1)$$

The cycle voltammetry (CV) measurement were carried out in 0.1 M KOH solution saturated with N_2 or O_2, ranging from 0.2 to 1.2V (vs RHE) at a rate of $50\ mV/s$. The linear scanning voltammetry (LSV) of the catalyst were tested by the rotating disk electrode (RDE) at different speeds of 400 to 2500 rpm and the scan rate is 10 mV/s. Finally the Kentucky-Lecich (K-L) spectrum is obtained by the corresponding RDE data, and the number of electron transfer during the ORR process is calculated by the K-L equation (equation (2) and (3))：

$$\frac{1}{J} = \frac{1}{J_K} + \frac{1}{B\omega^{1/2}} \qquad (2)$$

$$B = 0.62nFC_0(D_0)^{2/3}(\nu)^{-1/6} \qquad (3)$$

Where J represents the current density and J_K represents the kinetic-limiting current density in RDE, respectively. The ω represents the angular rate of the working electrode. Where n is the number of transfer electrons, f is the normal constant of Faraday (F = 96485 C mol^{-1}), C_0 (C_0 = 1.2*10^{-6} mol cm^{-3} for 0.1 M KOH) and D_0 (D_0 = 1.9*10^{-5} cm^2 s^{-1}) is a bulk and diffusion coefficient in 0.1 M KOH of O_2. And ν is the electrolyte motion viscosity (ν = 0.01 cm^2 s^{-1} in 0.1 M KOH). In addition, when rpm is a speed unit, a constant of 0.2 is applied.

The number of electron transfer during the inverse rate of the catalyst and the yield of hydrogen peroxide was measured in 0.1 M O_2-saturated KOH solution with a rotary annular disc electrode (RRDE) at 1600 rpm rotation rate and a scan rate of 50 mV/s.

Then, the n and H_2O_2 yields are calculated by the following formula (equation (4) and (5)):

$$n = 4 \times \frac{I_d}{I_d + I_r/N} \tag{4}$$

$$\%HO_2^- = 200 \times \frac{I_r/N}{I_d + I_r/N} \tag{5}$$

Where I_d is a disk current, I_r is a ring current. N (N = 0.37) is the current collection efficiency of the Pt ring.

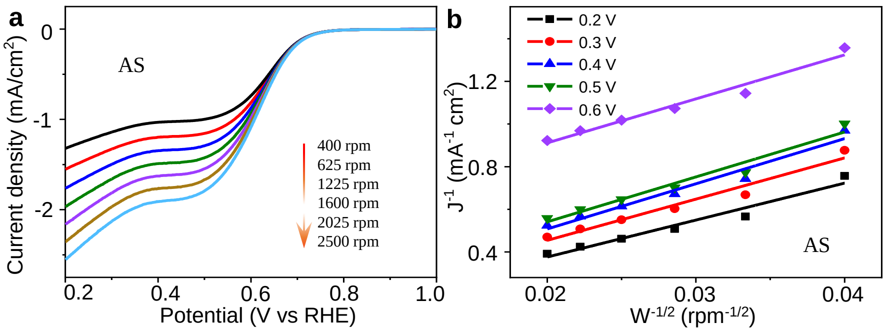

Figure S1 The LSV curves at different rotation rates from 400 to 2500 rpm of AS (a), corresponding to the K-L plots of AS from 0.2 to 0.6 V (b), respectively.

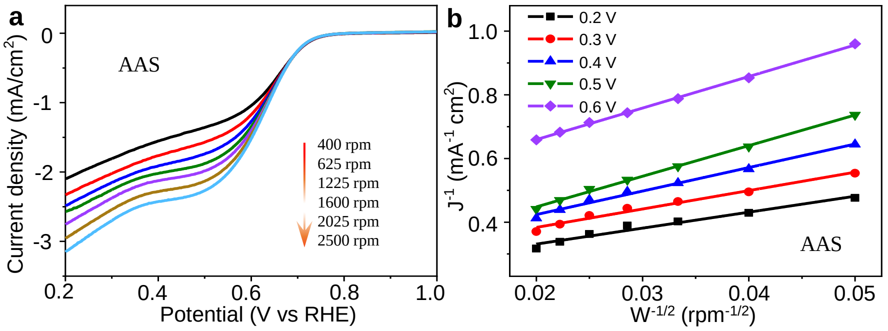

Figure. S2 The LSV curves at different rotation rates from 400 to 2500 rpm of AAS (a), corresponding to the K-L plots of AAS from 0.2 to 0.6 V (b), respectively.

Table S1. Synthesis process of catalyst AS, AAS and TAAS.

catalyst	Synthesis
AS	c

AAS	a and c
TAAS	a, b and c

a. Awn stem were activated in 1M KOH solution at 60 ℃ for 48 h;

b. Activated awn stem powder and trithiocyanuric acid were mixed in 50 mL of deionized water at a mass ratio of 1:3, stirred at room temperature for 2 h;

c. High temperature carbonization at 550 ℃ for 4 h in N_2 atmosphere and heated to 900 ℃ for 2 h.

Table S2. Physical properties and the charge transfer impedance of AS, AAS and TAAS.

Samples	S_{BET} (m^2 g^{-1})	Pore volume (cm^3 g^{-1})	Pore Size (nm)	I_D/I_G	R_{ct} (Ω)
AS	21.38	0.016	2.96	1.01	390
AAS	48.86	0.024	3.52	1.11	480
TAAS	191.04	0.13	2.59	1.18	550

Table S3. The content of C, N, O, and S elements and N configuration calculated of AS, AAS and TAAS from elemental analysis and XPS.

Samples	Elemental content (at. %)				N configuration (%)			
	C 1s	N 1s	S 2p	O 1s	pyridinic-N	pyrrolic-N	graphitic-N	oxidized-N
AS	87.02	1.47	0.22	11.29	16.0	36.5	36.1	11.3
AAS	88.25	1.34	0.82	9.59	17.8	34.8	34.5	12.9
TAAS	80.35	9.84	1.4	8.41	36.5	26.3	30.9	6.2
